# Real-world evaluation of attention deficit hyperactivity disorder symptoms and side effects in patients prescribed serdexmethylphenidate/dexmethylphenidate or other stimulants

**DOI:** 10.3389/fphar.2026.1769333

**Published:** 2026-05-21

**Authors:** Joel L. Young, Richard N. Powell, Anna Powell, Lisa L. M. Welling, Lauren Granata, Jaime Saal, Margot Nash, Meg Corliss

**Affiliations:** 1 Rochester Center for Behavioral Medicine, Rochester Hills, MI, United States; 2 MedaData, LLC, Rochester Hills, MI, United States; 3 School of Medicine - Wayne State University, Detroit, MI, United States; 4 Department of Psychology, Oakland University, Rochester, MI, United States; 5 Corium, LLC, Cambridge, MA, United States

**Keywords:** ADHD, duration, insomnia, long-acting, side effects

## Abstract

**Background:**

Serdexmethylphenidate/dexmethylphenidate (SDX/d-MPH) (Azstarys®) is an FDA-approved treatment for ADHD in patients aged 6 years and older. With its prodrug formulation, SDX/d-MPH provides rapid and sustained efficacy.

**Methods:**

This study compared the tolerability of SDX/d-MPH with other approved extended-release (ER) stimulant medications by evaluating patient-reported side effects in a real-world sample of patients with ADHD prescribed SDX/d-MPH, lisdexamfetamine (LDX), amphetamine ER (AMP), or methylphenidate ER (MPH). Digital survey responses from 1,395 patients (age: M ± SD = 32.5 ± 14.1 years, range = 7–78 years) treated for ADHD at a single large outpatient psychiatric practice were analyzed. Patients completed the ADHD Symptom and Side Effect Tracking (ASSET) scale in digital pre-visit surveys. Analyses focused on a filtered subset of 697 surveys reflecting early treatment (≤90 days) and ER stimulant monotherapy. Multinomial logistic regressions were conducted to examine associations between side effect frequency and prescription type. Differences in side effect frequency across prescription types were examined using one-way ANOVA with Tukey-adjusted comparisons and ANCOVA controlling for age and time since treatment initiation. To account for within-patient clustering, a MANCOVA including patient as a fixed effect was conducted as a robustness check.

**Results:**

Prescription type was associated with all four side effects (all *p* < 0.05). Logistic regression analyses indicated that insomnia (*p* = 0.006), end-of-dose crash (*p* = 0.046), and return of symptoms (*p* = 0.036) were associated with prescription type, although effect sizes were modest. Pairwise comparisons showed that patients taking SDX/d-MPH reported lower frequency of insomnia compared to AMP ER (*p* = 0.024) and LDX (*p* = 0.006), and lower frequency of end-of-dose crash compared to AMP ER (*p* = 0.046). MANCOVA found significant differences between prescription types were observed for insomnia (*p* = 0.036) and end-of-dose crash (*p* = 0.021), but not for poor appetite or return of symptoms.

**Conclusion:**

In this single-center retrospective analysis of patient surveys, patients with ADHD taking SDX/d-MPH reported reduced frequency of insomnia compared to AMP ER and LDX, and lower frequency of end-of-dose crash compared to AMP ER. This real-world tolerability analysis highlights the importance of considering side effects in ADHD treatment.

## 1 Introduction

Attention deficit hyperactivity disorder (ADHD) is a neurobehavioral disorder characterized by hyperactivity, impulsivity, and/or inattention (Young and Goodman, 2016; American Psychiatric Association, 2013). ADHD affects approximately 10% of children and adolescents in the United States, but can be diagnosed at any age (Li et al., 2023; Danielson et al., 2024). A 2024 survey study from the National Center for Health Statistics estimates 6% of adults in the United States currently have an ADHD diagnosis, with 55.9% receiving their diagnosis in adulthood (Staley et al., 2024). Untreated ADHD is associated with comorbid psychiatric disorders, substance use, and impaired functioning across social, academic, and occupational domains (Young, 2007; Weiss and Murray, 2003; Pawaskar et al., 2020). National guidelines recommend a combination of medication and behavioral treatments for children aged six and older, adolescents, and adults (Post and Kurlansik, 2012; Wolraich et al., 2019). Stimulant medications, including amphetamine and methylphenidate formulations, are shown to be safe and effective and have become a first-line treatment option for adults (Post and Kurlansik, 2012) and children (Danielson et al., 2024).

When treating ADHD, extended-release (ER) stimulants are preferred over immediate-release (IR) stimulants, which require frequent dosing and typically have poor adherence and lower patient satisfaction (Kolar et al., 2008; Marcus et al., 2005; Rothenberger et al., 2011). ER formulations generally have better tolerability, improved adherence, and reduced risk of abuse and diversion compared to IR formulations (Post and Kurlansik, 2012; Kolar et al., 2008; Marcus et al., 2005; Rothenberger et al., 2011; Childress, 2021; Wilens et al., 2008). Yet, even with ER medications, up to one-fourth of patients discontinue treatment due to inadequate symptom management or side effects (Schein et al., 2022; Khan and Aslani, 2019). Among healthcare providers, cost, perceived duration of effect, and side effects were considered the most important factors in deciding on a pharmacotherapy for ADHD patients (Adler et al., 2019).

Many once-daily methylphenidate medications include an immediate-release component and can maintain efficacy for up to 12 h after dosing (Kimko et al., 1999; Coghill et al., 2013). Individual variability and different time-action profiles of methylphenidate stimulants can lead to periods of poorer symptom control at certain times of day for some patients (Coghill et al., 2013). Developing treatments that can provide a balance between rapid onset and sustained, full-day symptom management while reducing side effects will better meet the needs of patients and expectations of providers.

Serdexmethylphenidate/dexmethylphenidate (SDX/d-MPH) (Azstarys®) is an FDA-approved treatment for ADHD in patients aged six and older (Author Anonymous, 2021). The approved and marketed version has a molar ratio of 70% SDX (a novel prodrug of d-MPH that is pharmacologically inactive until it is gradually converted to active d-MPH in the lower intestinal tract) and 30% d-MPH (Braeckman et al., 2022). The pharmacokinetic profile of SDX/d-MPH is characterized by a rapid increase in d-MPH, attributable to the d-MPH component, followed by sustained conversion of SDX to d-MPH to maintain extended action. This prodrug approach achieves a rapid onset and extended duration that may offer benefits over traditional ER d-MPH formulations (Braeckman et al., 2022; Barnhardt et al., 2023; Kollins et al., 2021). Efficacy of SDX/d-MPH was established in pediatric patients (6–12 years) in a randomized, double-blind, placebo-controlled, parallel group, analog classroom study, which demonstrated improved post-dose change from baseline in classroom behaviors with SDX/d-MPH compared to placebo (Author Anonymous, 2021; Kollins et al., 2021). Adolescent (13–17 years) and adult (18+ years) efficacy was supported by pharmacokinetic bridging between SDX/d-MPH (52.3 mg/10.4 mg) and dexmethylphenidate hydrochloride ER capsules (Author Anonymous, 2021).

Despite promising pharmacological properties, there is limited published real-world evidence describing side effects in patients prescribed ER treatments for ADHD, including SDX/d-MPH. Given the prodrug design of SDX/d-MPH produces a more gradual rise in d-MPH exposure, it may reduce peak-related adverse effects and therefore offer improved tolerability compared to other long-acting amphetamine and methylphenidate formulations. This study aimed to evaluate patient-reported side effects of pharmacotherapy in a real-world sample of patients prescribed SDX/d-MPH (Azstarys®), lisdexamfetamine (Vyvanse®), amphetamine ER (Adderall® XR or generic ER amphetamine formulations), or methylphenidate ER (Concerta®, Focalin® XR, or generic methylphenidate ER/long-acting formulations). The sample was drawn from patients who received treatment at a large outpatient psychiatric center, which administers an ADHD side effect questionnaire as part of their standard care (Young et al., 2023). This single-center approach provides the benefit of consistent side effect tracking for all patients.

## 2 Methods

### 2.1 Study design and data retrieval

This was a retrospective review of digital survey responses, archived with time-point specific snapshots of patient chart information, including recent prescribing activity acquired from medical treatment records for individuals treated at the Rochester Center for Behavioral Medicine (RCBM) in Rochester Hills, MI, a large outpatient psychiatric practice consisting of 30 psychiatric professionals. All methods were performed in accordance with the ethical standards laid down in the 1964 Declaration of Helsinki and its later amendments. All participants involved in this study provided informed consent and gave permission for their de-identified data to be used in undisclosed future research and publication prior to their participation, and secondary research use of their information was deemed ethical by the responsible IRB (approval number: IRB-FY2024–242).

Survey responses were retrieved from RCBM via their HIPAA-compliant Qualtrics platform, which securely administers patient-facing forms, questionnaires, and psychometric assessments. Pharmacy data was transmitted through the practices’ electronic medical records system. Data used in analyses were de-identified such that no items in the extracted data sheet contained any of the 18 identifiers described in the HIPAA privacy rule’s safe harbor provision, ensuring that, to the furthest extent possible, no reidentification of any patients would be possible.

### 2.2 Procedures

Patients initiated treatment at RCBM either via self-referral or referral from a healthcare provider. Before their intake appointment and each subsequent appointment, patients completed a pre-visit survey through Qualtrics, an online survey distribution software integrated with RCBM’s electronic charting program through the Qualtrics API. This system connects the patients’ data with their medical history and clinician assessments for the clinician to review prior to each visit. RCBM’s database was filtered to acquire patient records meeting study eligibility per the inclusion criteria. The digital pre-visit survey includes the ADHD Symptom and Side Effect Tracking Scale (ASSET) (Young et al., 2023), a validated assessment measuring the magnitude of impact of ADHD symptoms on daily life resulting in a weighted factor score between 1 and 6 with a clinical cut-off score of 4.40 indicating rationale for further clinical assessment for ADHD. The ASSET is accompanied by a side effect tracking questionnaire, where patients indicate the frequency with which they experience common side effects (insomnia, generalized pain, fatigue, dry mouth, poor appetite, food binges, tics, anger, suspiciousness, restless legs, end-of-dose crash, unwanted changes in weight, and return of symptoms). The side effect list was derived from DSM-IV criterion and updated in accordance with the DSM-5 (Young et al., 2023). The side effect items reflect patient-reported experiences during ongoing treatment and do not distinguish between pre-existing symptoms and treatment-emergent effects. Patients rated each item on a five-point scale (0 = never, 1 = rarely, 2 = occasionally, 3 = often, 4 = always).

### 2.3 Participants

Survey responses were retrieved for patients with psychiatric office visits occurring between May 2022 and June 2024. Eligible surveys were completed by patients with a diagnosis of ADHD, as indicated by clinician-assigned ICD-10 codes documented in their medical record, who were prescribed lisdexamfetamine (LDX) (Vyvanse), amphetamine (AMP) ER (Adderall XR and generic ER amphetamine formulations), methylphenidate (MPH) ER or d-methylphenidate (d-MPH) ER (Concerta, Focalin XR, or generic methylphenidate ER/d-MPH ER formulations), or SDX/d-MPH (Azstarys) as monotherapy within the 30 days before the survey date. Surveys were excluded if the patient was also prescribed an immediate-release stimulant or a non-stimulant for ADHD, or if there were fewer than 28 days between their last two prescription dates. These criteria were applied to improve comparability across treatment groups and to isolate side effect reporting associated with a single extended-release stimulant. However, comprehensive treatment history prior to the observation period was not available, so it was not possible to determine whether patients were stimulant-naïve or had switched from other medications prior to the study period.

A total of 1,395 patients (age: M ± SD = 32.5 ± 14.1 years, range = 7–78 years) met inclusion criteria, comprising 96 prescribed SDX/d-MPH, 158 prescribed MPH ER, 64 prescribed d-MPH ER, 380 prescribed amphetamine ER, and 697 prescribed lisdexamfetamine for ADHD, across a range of doses. The sample was 57% female, 39% male, and 4% non-cisgender. Of the 1,395 patients, 87.2% were white, 3.1% were Asian, 2.6% were Black/African American, 1.3% were Native American or Pacific Islander, and 5.7% declined to provide their ethnicity. Of the full sample, 13.7% (*n* = 191) were children/adolescents (<18 years) and 86.3% (*n* = 1,204) were adults (≥18 years). Sample demographics are shown in Table 1.

**TABLE 1:** Patient demographics (*N* = 1395).

Participants	Age in years, mean ± SD (range)
Full sample mean ± SD	32.5 ± 14.1 (7–78)
Children (<18 years) mean ± SD	14.12 ± 2.44
Adults (≥18 years) mean ± SD	35.44 ± 12.87

Gender	%
Female	57
Male	39
Non-cisgender	4

Race	%
White/Caucasian	87.2
Asian	3.1
Black/African American	2.6
Native American or Pacific Islander	1.3
Declined to provide race	5.7

### 2.4 Statistical analysis

This study assessed four side effects: insomnia, poor appetite, end-of-dose crash, and return of symptoms as dose wears off. Insomnia and poor appetite are common treatment-related complications associated with treatment discontinuation or change (Schein et al., 2022), and end-of-dose crash and return of symptoms are most related to duration of action.

#### 2.4.1 Data filtering and analytic sample

Prescriptions associated with surveys completed within the first 90 days of the patient’s first office visit were presumed to be their first-line treatment, although comprehensive treatment history prior to the observation period was not available for this dataset. Therefore, the sample was filtered to only include surveys completed within this time frame and surveys completed at a time when more than one prescription had been sent in the last 30 days were excluded to control for polypharmacy impacts on tolerability survey items. After filtering, only 18 surveys were completed by patients prescribed d-MPH ER (Focalin XR or generic equivalent), so this group was excluded from subsequent analyses because the sample size was inadequate. The final sample included 697 surveys from patients prescribed ER stimulant monotherapy: 296surveys from patients taking AMP ER, 223 from patients taking LDX, 114 from patients taking MPH ER, and 64 from patients taking SDX/d-MPH.

#### 2.4.2 Covariate assessment

To inform covariate selection, Pearson’s correlations were performed to identify associations between potential covariates and side effect outcomes, including the number of surveys completed per patient to assess potential non-independence of observations. A correlation matrix was calculated with the variables: frequency of insomnia, frequency of poor appetite, frequency of end-of-dose crash, frequency of return of symptoms as dose wears off, patient age, number of surveys belonging to the patient, and number of days since the patient began psychiatric treatment (days since first prescription date). Covariate selection for subsequent models was informed by both these associations and conceptual relevance, with age and time since treatment initiation included given their established relationships with stimulant metabolism, side effect profiles, and treatment exposure (Graham and Coghill, 2008; Sibley et al., 2022; Arnold et al., 2015).

#### 2.4.3 Multinomial logistic regression analyses and unadjusted group comparisons

To determine whether prescription type could be predicted by side effect frequency, logistic regressions were conducted for each side effect and McFadden’s *R* ^*2*^ was used to assess goodness of fit for the model. Specifically, logistic regression models were used to examine whether patient-reported side effect frequency was associated with prescription type. Given the observational nature of the data and non-randomized prescription type assignment, these analyses are descriptive of associations and do not support causal inference regarding medication effects. Next, side effect frequencies were also compared across prescription types using one-way ANOVA with Tukey-adjusted *post hoc* pairwise comparisons between each prescription type.

#### 2.4.4 Adjusted group comparisons and multivariate analysis accounting for within-patient clustering

Analysis of covariance (ANCOVA) was used to test for differences in side effect frequency across prescription types while controlling for age and days since treatment initiation. To further address potential non-independence of observations due to repeated surveys, a multivariate analysis of covariance (MANCOVA) was conducted across all four side effects, with patient ID included as a fixed effect to account for between-subject variability. This analysis was conducted as a robustness check to evaluate whether the overall pattern of results remained consistent when accounting for within-patient clustering.

#### 2.4.5 Sex differences and sex × prescription type interactions

Exploratory analyses tested age × prescription type interactions to examine whether associations between age and side effects differed by prescription type. Multiple statistical tests were conducted across side effects and prescription types, and results should thus be interpreted with consideration of the increased risk of Type I error.

## 3 Results

### 3.1 Covariate assessment

Pearson’s correlations found no significant relationship between the number of surveys a patient completed and frequency of insomnia (*r* = 0.072, *p* = 0.067) or end-of-dose crash (*r* = −0.037, *p* = 0.376), though it was positively associated with poor appetite (*r* = 0.190, *p* < 0.001) and negatively associated with return of symptoms (*r* = −0.161, *p* < 0.001). Age was negatively correlated with the frequency of experiencing poor appetite (*r* = −0.086, *p* = 0.030) and end-of-dose crash (*r* = −0.082, *p* = 0.047), and positively associated with insomnia (*r* = 0.101, *p* = 0.011), but was not associated with return of symptoms (*r* = 0.006, *p* > 0.88), indicating that younger patients reported higher frequency of poor appetite and end-of-dose crash, and lower levels of insomnia. The number of days since the patient began psychiatric treatment was negatively correlated with the frequency of experiencing poor appetite (*r* = −0.093, *p* = 0.018), but not with insomnia *(r* = −0.071, *p* = 0.073), end-of-dose crash (*r* = 0.004, *p* = 0.923), or return of symptoms (*r* = −0.009, *p* = 0.823). Among side effects, end-of-dose crash and return of symptoms were strongly correlated (*r* = 0.515, *p* < 0.001).

### 3.2 Multinomial logistic regression analyses and unadjusted group comparisons

**FIGURE 1:**
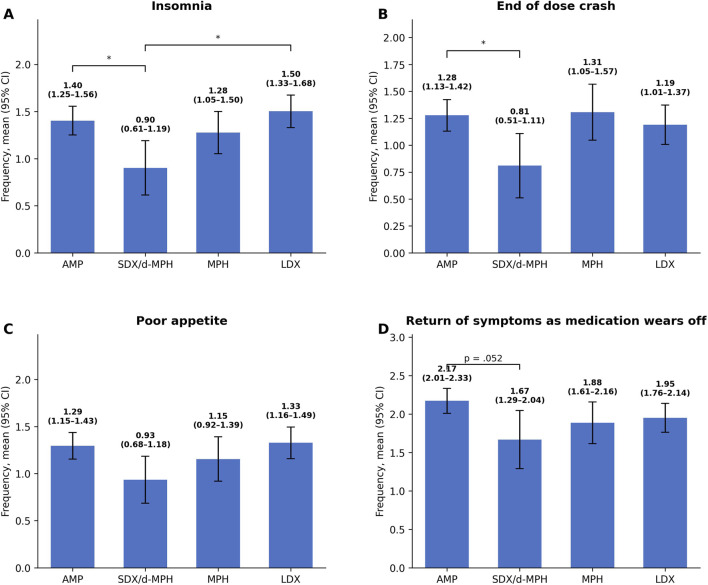
Mean frequency of reported side effects by prescription type. Panels show **(A)** insomnia, **(B)** end-of-dose crash, **(C)** poor appetite, and **(D)** return of symptoms as the dose wears off. Means and 95% CIs are presented for each medication group. Asterisks (*) indicate significant differences. Error bars represent standard error. Significant differences between treatment groups were observed for insomnia and return of symptoms, but not for poor appetite or end-of-dose crash at the omnibus level, although a significant pairwise difference was observed for end-of-dose crash between SDX/d-MPH and AMP ER.

Multinomial logistic regression, patients prescribed SDX/d-MPH reported a lower total number of the four indicated that side effect frequency was associated with prescription type for insomnia (*p* = 0.006, McFadden’s *R* ^*2*^ = 0.008), end-of-dose crash (*p* = 0.046, McFadden’s *R* ^*2*^ = 0.005), and return of symptoms (*p* = 0.036, McFadden’s *R* ^*2*^ = 0.006), but not for poor appetite (*p* = 0.093, McFadden’s *R* ^*2*^ = 0.004). One-way The ANOVA found that frequency of insomnia (*F*_3, 639_ = 3.92, *p* = 0.009*p = 0.005*; Figure 1A) and return of symptoms (*F*_3, 590_ = 2.87, *p* = 0.036), but not poor appetite (*F*_3, 641_ = 2.05, p > 0.10) or end-of-dose crash (*F*_3, 586_ = 2.52, *p* > 0.05; see Figure 1). Pairwise comparisons showed that patients taking SDX/d-MPH had the lowest frequency of insomnia compared to AMP (*p* = 0.02409) and LDX (*p* = 0.0036). For end-of-dose crash, SDX/d-MPH was associated with lower frequency compared to AMP ER (*p* = 0.046). Differences in return of symptoms between SDX/d-MPH and AMP ER approached significance (*p* = 0.052; Figure 1).

### 3.3 Adjusted group comparisons

ANCOVA analyses controlling for age and days since the start of psychiatric treatment revealed significant effects of prescription type for all four side effects: insomnia (*F*_3, 642_ = 3.24, *p* = 0.022), poor appetite (*F*_3, 644_ = 3.44, *p* = 0.017), end-of-dose crash (*F*_3, 589_ = 3.36, *p* = 0.019), and return of symptoms (*F*_3, 595_ = 2.97, *p* = 0.032). To test whether the relationship between age and side effects varied across prescription types, age × prescription type interaction terms were included in the ANCOVA models for each side effect. Where significant interactions were observed, follow-up Pearson correlations between age and the relevant side effect were conducted within each prescription type to characterize the nature of the interaction. These exploratory analyses revealed a significant age × prescription type interaction for insomnia (*p* < 0.001), but not for poor appetite (*p* > 0.14), end-of-dose crash (*p* > 0.50), or return of symptoms (*p* > 0.65). Follow-up analyses indicated that age was positively associated with insomnia among patients prescribed AMP ER (*r* = 0.206, *p* = 0.001) and showed a similar nonsignificant trend for LDX (*r* = 0.127, *p* = 0.069), whereas age was negatively associated with insomnia among patients prescribed SDX/d-MPH (*r* = −0.331, *p* = 0.009). No significant association was observed for MPH ER (*r* = −0.072, *p* > 0.45).

### 3.4 Multivariate analysis accounting for within-patient clustering

To account for within-subject clustering, a MANCOVA including all four side effects and patient ID as a fixed effect was conducted. This analysis revealed a significant multivariate effect of prescription type (Wilks’ Λ = 0.886, *F*_12, 529.44_ = 2.07, *p* = 0.018), indicating overall differences in side effect profiles across prescription types after accounting for between-patient variability. Follow-up univariate tests indicated significant effects of prescription type for insomnia (*F*_3, 203_ = 2.90, *p* = 0.036) and end-of-dose crash (*F*_3, 203_ = 3.31, *p* = 0.021), whereas effects for poor appetite (*F*_3, 203_ = 1.11, *p* > 0.34) and return of symptoms (*F*_3, 203_ = 2.06, *p* = 0.107) were not significant.

### 3.5 Sex differences and sex × prescription type interactions

Finally, exploratory analyses examined sex differences in side effect frequency across prescription types. Independent samples *t*-tests indicated that females reported higher overall frequency of poor appetite than males (*t* = 2.56, *p* = 0.011, *d* = 0.21), whereas no significant sex differences were observed for insomnia (*t* = −0.03, *p* > 0.97), end-of-dose crash (*t* = 0.72, *p* > 0.47), or return of symptoms (*t* = 0.39, p > 0.70). To further examine whether sex moderated associations between prescription type and side effects, sex × prescription type interactions were tested within ANCOVA models controlling for age and days since treatment initiation. A significant interaction was observed for poor appetite (*F*_3, 633_ = 5.30, *p* = 0.001), whereas interactions were not significant for other side effects (all *p* > 0.45). Follow-up analyses indicated that the interaction for poor appetite was driven by a significant sex difference within the amphetamine ER group, with females reporting higher levels than males (*t* = 4.47, *p* < 0.001, *d* = 0.56). No significant sex differences were observed within the SDX/d-MPH, MPH ER, or LDX groups (all *p* > 0.10).

## 4 Discussion

In this single-center retrospective analysis of patient records, patients with ADHD taking SDX/d-MPH reported reduced frequency of certain side effects compared to those taking AMP ER, MPH ER, or LDX ER stimulant treatments. Using the side effect tracking scale accompanying the ASSET (Young et al., 2023), a validated ADHD assessment tool, significant effects of ER prescription type were observed across multiple analytic approaches. In survey-level ANCOVA models, prescription type was associated with all four side effects. However, when accounting for within-patient clustering using a MANCOVA with patient as a fixed effect, the multivariate omnibus test was significant, and univariate effects remained significant for insomnia and end-of-dose crash, but not for poor appetite or return of symptoms. Converging evidence across these approaches suggests that differences in insomnia and end-of-dose crash are the most robust findings. These findings extend prior research by providing real-world data on patient-reported tolerability. Despite the widespread use and well-described safety profile of stimulants as first-line treatments for ADHD, all stimulant treatments are associated with side effects of varying severity (Graham and Coghill, 2008; Wu et al., 2025; Cortese et al., 2018). Clinicians weigh the comparative benefits and risks of different medications when making treatment decisions such as dose changes and treatment switches. However, head-to-head comparisons of tolerability are lacking for newer ADHD treatments, and few studies statistically compare individual side effects (Coghill et al., 2013; Miller-Horn et al., 2015; Childress et al., 2023). Results of the present study show SDX/d-MPH was associated with lower reported frequency of side effects related to insomnia compared to AMP ER, MPH ER, and LDX ER, and lower reported frequency of experiencing a “crash” at the end of a dose as it wears off compared to AMP ER. These differences were observed consistently across analytic approaches that accounted for covariates and, in the case of the MANCOVA, within-patient clustering. With more data comparing efficacy and tolerability of approved drugs, a new modified stimulant, such as SDX/d-MPH, could become a useful option for treating ADHD in pediatric and adult patients where tolerability is particularly important.

Evaluating the effects of prescription type on insomnia in patients with ADHD is challenging because sleep disturbance is a common symptom of ADHD and a potential side effect of all stimulants (Mattingly et al., 2023; Stein et al., 2012). In adults, insomnia also correlates with severity of ADHD symptoms, highlighting the importance of reducing the overall impact of insomnia when deciding between treatments (Hvolby, 2015). Importantly, the present study did not include baseline assessments of sleep prior to medication initiation. Accordingly, reported insomnia may reflect a combination of pre-existing sleep disturbances associated with ADHD and treatment-related side effects, and differences in reported insomnia should be interpreted as differences in patient-reported sleep disturbance during treatment rather than definitive evidence of differential causal effects of medications on sleep. However, similar to the results shown here, a study of SDX/d-MPH in children showed less severe sleep disturbances and sleep consistent with pre-treatment patterns after one year of SDX/d-MPH treatment compared to placebo (Mattingly et al., 2023). MPH without a prodrug is associated with sleep disturbances (Sangal et al., 2006; Faraone et al., 2019), but effects depend on whether the patient experienced sleep disturbances prior to treatment (Kim et al., 2010; Becker et al., 2016). A meta-analysis of 35 studies of sleep disturbances with MPH in youth with ADHD found an impact of MPH on sleep-related adverse events, particularly for ER MPHs (Faraone et al., 2019), although individual clinical trials show conflicting results depending on specific characteristics of patients and the type of disturbance measured (Stein et al., 2012; Sangal et al., 2006; Sobanski et al., 2008). For example, a placebo-controlled trial of MPH found that patients took longer to fall asleep and slept less overall, but experienced more periods of uninterrupted sleep (Boonstra et al., 2007). More specific descriptions of insomnia patterns and longitudinal research incorporating pre-treatment sleep measures in future studies will provide more informative insights into the effects of SDX/d-MPH on sleep.

Amphetamines and methylphenidates are both associated with a “crash” in the afternoon or evening as a symptom of the dose wearing off, thought to be attributable to sudden imbalances in intracellular and synaptic dopamine after repeated stimulant exposure (Breggin, 1999). This may lead to a temporary increase in ADHD symptoms and tiredness, irritability, restlessness, impulsivity, and reduced concentration, commonly reported in classroom studies in the afternoon in children when doses begin to wear off (Kooij, 2011; Kollins et al., 2021; Childress et al., 2020; Wigal et al., 2013). The characteristic stimulant crashes are often described as fatigue, brain fog, irritability, and changes in mood that are not associated with the patient’s typical ADHD symptoms, while the return of symptoms can mimic the experience of unmedicated ADHD, sometimes to an exaggerated degree (Kooij, 2011; Kollins et al., 2021). In the present study, SDX/d-MPH was associated with lower reported frequency of end-of-dose crash relative to AMP ER, a finding that remained significant in both ANCOVA and MANCOVA models. In contrast, differences in return of symptoms were less consistent across analytic approaches and did not remain significant when accounting for within-patient clustering. Experiencing a “crash” may be a more salient or distinct subjective experience to patients than the gradual return of ADHD symptoms, which may influence reporting. More in-depth surveys of elements relevant to medication wearing-off effects may provide greater insight into patients’ experiences at the end of a dose (Oppenh et al., 2019; Gringras et al., 2006). Describing side effects at different times of day have important implications for understanding the unique treatment durations and wear-off effects of different ER stimulants. Future work incorporating more fine-grained temporal assessment of symptom rebound and wear-off effects would help disentangle these related constructs.

Exploratory analyses provided limited evidence for sex-related variation in side effect reporting. Females reported higher overall frequencies of poor appetite than males, whereas no significant sex differences were observed for insomnia, end-of-dose crash, or return of symptoms. A significant sex × prescription type interaction was observed for poor appetite, driven by a difference within the amphetamine group that was not evident for other prescription types. These findings provide limited support for prior work suggesting sex-related variation in stimulant response and tolerability (Rapoport and Groenman, 2025; Müller and Fender, 2026), and should be interpreted cautiously given the exploratory nature of these analyses and the unequal sex distribution in the sample. Future research using designs specifically powered to examine sex as a moderator will be important to clarify these patterns.

The current sample spanned a wide age range (7–78 years), which enhances generalizability, but also introduces developmental heterogeneity. In the present analyses, age was associated with multiple side effects, and a significant age × prescription type interaction was observed for insomnia. Older age was associated with higher insomnia among AMP ER patients and, to a lesser extent, LDX patients, whereas the opposite pattern was observed for SDX/d-MPH patients. These results suggest that age-associated tolerability may vary across stimulant formulations. Although age was included as a covariate in all statistical models, this approach only accounts for linear age-related differences and does not fully capture developmental variation in stimulant response and side-effect profiles (Graham and Coghill, 2008). Because some medication groups were not sufficiently powered to examine age-stratified effects, future research should directly compare pediatric and adult populations using stratified or longitudinal designs to better characterize age-specific tolerability profiles.

This analysis is further limited by sample size imbalances across prescription types, particularly the relatively small number of patients prescribed SDX/d-MPH after filtering. Although this distribution reflects real-world prescribing patterns during the study period because SDX/d-MPH is a newer medication, it may have limited statistical power and the stability of estimates for this group. Accordingly, observed differences should be interpreted cautiously. Replication in larger, more balanced samples and using prospective designs will be important to confirm these findings. Additionally, samples included a range of different dosages, and treatments at different dosages were not compared. The effective dose of a medication is individually variable, and dose reductions are recommended if the patient experiences unmanageable side effects (Graham and Coghill, 2008; Graham et al., 2011). Thus, patients requiring higher doses to achieve efficacy may experience more severe side effects (Fredriksen et al., 2014; Sachdev and Trollor, 2000). It is possible that comparing treatments within dosages would yield different results, but the current sample was underpowered to detect dose- and treatment-based effects. Future research should further investigate possible treatment and dose interactions on reported side effects in ADHD patients.

The analytic approach also has important limitations. Analyses were restricted to early treatment periods to reduce repeated observations and approximate early treatment exposure. However, comprehensive treatment history prior to the observation period was not available, and it was therefore not possible to determine whether patients were stimulant-naïve or had switched from other medications. As a result, some patients may have initiated treatment prior to entering the study window or changed medications during care. These factors may further influence both side effect reporting and treatment selection. Modeling prescription type as the outcome also does not allow for causal inference regarding the effects of medications on side effects. As this was a non-randomized observational study, prescription groups may differ on unmeasured factors not captured in the dataset. Although analyses adjusted for age and time since treatment initiation, unmeasured confounding variables, including baseline ADHD symptom severity and prior treatment history, may influence both treatment selection and reported side effects. Survey-level analyses did not indicate strong associations between the number of responses per patient and most side effects, but repeated observations introduce potential non-independence issues. The inclusion of a MANCOVA with patient as a fixed effect partially addresses this issue by accounting for between-subject variability, and the convergence of findings across analytic approaches strengthens confidence in the primary results. Nevertheless, as the dataset does not fully account for within-subject dependence, some degree of non-independence cannot be entirely ruled out. Multiple statistical comparisons were also conducted, increasing the possibility of Type I error. Accordingly, the present findings should be interpreted as exploratory and hypothesis-generating. Finally, the McFadden *R* ^2^ values for the logistic models were modest, indicating that a large proportion of variability in side effects remains unexplained. This suggests that additional clinical or contextual factors likely contribute to side-effect burden and should be examined in future work.

Overall, the results of this study show lower reported frequency of side effects with SDX/d-MPH compared to other ER stimulants, particularly insomnia and end-of-dose crash, with these effects showing the greatest consistency across analytic approaches that account for covariates and within-patient clustering. The results highlight the importance of considering side effects when making treatment decisions for ADHD and add to prior research describing sleep and end-of-dose effects with SDX/d-MPH (Barnhardt et al., 2023; Mattingly et al., 2023).

## Data Availability

The raw data supporting the conclusions of this article will be made available by the authors, without undue reservation.
